# Glutaminase inhibition in combination with azacytidine in myelodysplastic syndromes: Clinical efficacy and correlative analyses

**DOI:** 10.21203/rs.3.rs-2518774/v1

**Published:** 2023-02-23

**Authors:** Marina Konopleva, Courtney DiNardo, Tushar Bhagat, Natalia Baran, Alessia Lodi, Kapil Saxena, Tianyu Cai, Xiaoping Su, Anna Skwarska, Veronica Guerra, Vinitha Kuruvilla, Sergej Konoplev, Shanisha Gordon-Mitchell, Kith Pradhan, Srinivas Aluri, Meghan Collins, Shannon Sweeney, Jonathan Busquet, Atul Rathore, Qing Deng, Michael Green, Steven Grant, Susan Demo, Gaurav Choudhary, Srabani Sahu, Beamon Agarwal, Mason Spodek, Victor Thiruthuvanathan, Britta Will, Ulrich Steidl, George Tippett, Jan Burger, Gautam Borthakur, Elias Jabbour, Naveen Pemmaraju, Tapan Kadia, Steven Komblau, Naval Daver, Kiran Naqvi, Nicholas Short, Guillermo Garcia-Manero, Stefano Tiziani, Amit Verma

**Affiliations:** The University of Texas, MD Anderson Cancer Center; UT MD Anderson Cancer Center; Albert Einstein College of Medicine; The University of Texas MD Anderson Cancer Center; College of Natural Sciences, The University of Texas at Austin; The University of Texas, MD Anderson Cancer Center; The University of Texas, MD Anderson Cancer Center; Dan L. Duncan Cancer Center and , Baylor College of Medicine; Albert Einstein College of Medicine-Montefiore Medical Center; MD Anderson Cancer Center; The University of Texas, MD Anderson Cancer Center; MD Anderson cancer Center, The University of Texas; Albert Einstein College of Medicine; Albert Einstein College of Medicine; Albert Einstein College of Medicine; College of Natural Sciences, The University of Texas at Austin; Department of Nutritional Sciences, Dell Pediatric Research Institute, Dell Medical School, The University of Texas at Austin, Austin, TX, USA; Dell Medical School, The University of Texas at Austin; Dell Medical School, The University of Texas at Austin; The University of Texas MD Anderson Cancer Cent; MD Anderson Cancer Centre; Department of Medicine, Virginia Commonwealth University; Calithera Biosciences; Albert Einstein College of Medicine-Montefiore Medical Center; Albert Einstein College of Medicine; GenomeRxUs LLC; Albert Einstein College of Medicine-Montefiore Medical Center; Albert Einstein College of Medicine; Albert Einstein College of Medicine; Albert Einstein College of Medicine; The University of Texas, MD Anderson Cancer Center; MD Anderson Cancer Center; The University of Texas MD Anderson Cancer Center; The University of Texas MD Anderson Cancer Center; The University of Texas, MD Anderson Cancer Center; The University of Texas MD Anderson Cancer Center; The University of Texas MD Anderson Cancer Center; The University of Texas MD Anderson Cancer Center; The University of Texas, MD Anderson Cancer Center; The University of Texas, MD Anderson Cancer Center; MD Anderson Cancer Center; Department of Nutritional Sciences, Dell Pediatric Research Institute, Dell Medical School, The University of Texas at Austin, Austin, TX, USA; Montefiore Einstein Cancer Center

## Abstract

Malignancies can become reliant on glutamine as an alternative energy source and as a facilitator of aberrant DNA methylation, thus implicating glutaminase (GLS) as a potential therapeutic target. We demonstrate preclinical synergy of telaglenastat (CB-839), a selective GLS inhibitor, when combined with azacytidine (AZA), *in vitro* and *in vivo*, followed by a phase Ib/II study of the combination in patients with advanced MDS. Treatment with telaglenastat/AZA led to an ORR of 70% with CR/mCRs in 53% patients and a median overall survival of 11.6 months. scRNAseq and flow cytometry demonstrated a myeloid differentiation program at the stem cell level in clinical responders. Expression of non-canonical glutamine transporter, SLC38A1, was found to be overexpressed in MDS stem cells; was associated with clinical responses to telaglenastat/AZA and predictive of worse prognosis in a large MDS cohort. These data demonstrate the safety and efficacy of a combined metabolic and epigenetic approach in MDS.

## Introduction

Cancer cells exploit a variety of metabolic pathways to fuel tumor expansion. Altered metabolic profiles, now recognized as a key hallmark of cancer, can promote rapid malignant growth and proliferation, as well as augment tumor survival in a hypoxic environment.^1–3^

While solid tumors rely primarily on aerobic glycolysis for energy production,^4^ hematological malignant cells develop a strong dependency on oxidative phosphorylation utilizing mostly glutamine. Glutamine is the most abundant amino acid present in plasma, and tumor cells often depend on glutamine availability for glutaminolysis, the mitochondrial pathway whereby glutamine is hydrolyzed by the glutaminase enzyme to glutamate. Glutamate can then be directly converted into α-ketoglutarate (α-KG) to fuel both the tricarboxylic acid (TCA) cycle, and to supply the antioxidant glutathione (GSH).^1^ This “glutamine addiction” is well characterized in certain cancers including glioblastoma, triple-negative breast cancer, renal cell cancer, and several hematologic malignancies such as multiple myeloma, acute lymphoblastic leukemia, acute myeloid leukemia (AML) and the myelodysplastic syndromes (MDS).^5–8^

Glutaminase (GLS) is a mitochondrial enzyme catalyzing the first step in glutamine metabolism. GLS is upregulated in glutamine-dependent tumors and thus represents a potential tumor vulnerability and promising therapeutic target. Telaglenastat (CB-839) is a first-in-class potent and selective allosteric small molecule glutaminase inhibitor, which has demonstrated *in vitro* and *in vivo* activity against multiple glutamine-dependent tumors.^5,7,9–11^ We reported high mRNA levels of the GLS splicing variant *GAC* in high-risk AML with complex cytogenetics ^10^, and previously showed that treatment with CB-839 interfered with the citric acid cycle, reduced the NADH/NAD^+^ ratio and ATP levels, inhibited cell proliferation and viability, and reduced the basal and maximal respiratory capacities [oxygen consumption rate (OCR)]^12,13^. As a single agent, increasing doses of CB-839 resulted in glutaminase inhibition in platelets and in tumors, with 800 mg BID representing the highest safe and well-tolerated monotherapy dose.^14^

MDS represents a malignant clonal hematopoietic disorder characterized by ineffective hematopoiesis, bone marrow (BM) dysplasia, peripheral cytopenias, and a propensity to transform into AML.^15,16^ Currently, the hypomethylating agents (HMA) azacytidine (AZA) or decitabine are the standard of care for patients with higher-risk MDS, and work to improve cytopenia, decrease transfusion requirements, extend patient survival, and reduce the risk of AML progression.^17,18^ However, only a minority of patients experience remission or durable hematologic improvements with HMA therapy, and outcomes at the time of HMA failure are dismal.^19,20^ Especially when considering the advanced age of most patients with MDS, improved and non-cytotoxic treatment options are needed. In this study, we demonstrate high expression of GLS and glutamine transporter SLC38A1 in poor risk MDS, and additive growth-inhibitory activity of CB-839 when combined with AZA, secondary to reduced glutamine consumption. Of importance, single cell transcriptomic and flow cytometry studies demonstrated a myeloid differentiation program at the stem cell level in clinical responders. We designed and herein report the safety and clinical activity of the combination of telaglenastat with AZA for patients with advanced MDS.

## Results

GLS is overexpressed in high risk MDS stem and progenitor cells and exists mainly as shorter GAC isoform in leukemic cells

**Figure 1:**
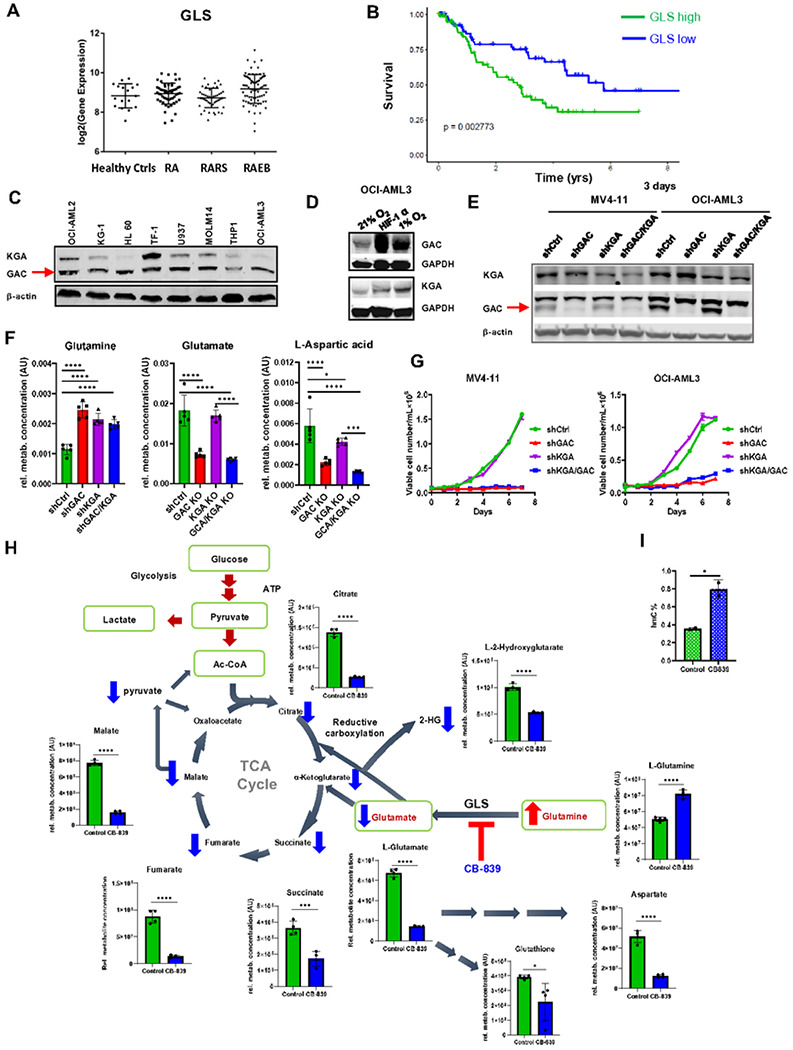
GLS is overexpressed in RAEB subtype of MDS and in subsets of AML and is associated with worse prognosis: **(A)** GLS expression in 183 cases of MDS and 17 control marrow CD34^+^ stem cells are shown. RAEB (refractory anemia with excess blasts, *p* <0.05), RA (Refractory anemia), RARS (Refractory anemia with ringed sideroblasts). **(B)** Kaplan Meier curves show worse survival of MDS patients with higher expression of GLS (Log rank *p =0.003*). **(C)** GAC isoform of GLS in expressed in AML cell lines. **(D)** GAC isoform of GLS protein is upregulated in leukemia cells over-expressing HIF-1α (middle lane) or cultured under hypoxic 1%O_2_ conditions (right). **(E)** Stable transfected OCI-AML3 cells with generated using shRNA control, puro-GAC plasmid, puro-KGA plasmid, puro-D3 (both isoforms) plasmid with puromycin incubation (3 days, 1 μg/ml). The expression of GAC and KGA in MV4;11 or OCI-AML3 cells was analyzed by Western blot. **(F)** LCMS analysis of OCIAML3 subjected to inducible knockdown of GAC, KGA or both GAC/KGA. GAC KO and less KGA KO drives metabolic perturbations in AML cell lines as shown as changes in level of glutamine, glutamate, and aspartate; (mean ± SD; n=5 replicates). **(G)** Effects of GAC, KGA and combined GAC/KGA knockdown were assessed on leukemic cells viability in MV4-11 and OCI-AML3 cells. Cell numbers for each cell line in indicated days were counted by Vi-cells. (t-test, mean ± SD, n=3 replicates). ***p ≤ 0.001 **(H)** LCMS analysis of selected metabolites extracted from HL60 cell lines, treated with vehicle or telaglenastat (CB-839) 1 μM, 24 hr (mean ± SD, n=4 replicates). **(I)** % of hypomethylation measured in OCIAML3 cells subjected to treatment with DMSO or CB-839 after 72 hours, (mean ± SD of n=3 replicates).

We determined GLS expression in transcriptomes derived from 183 MDS BM CD34 + stem and progenitor cells and compared them to 17 healthy controls ^21^. GLS was significantly increased in patients with refractory anemia with excess of blasts (RAEB), the MDS subtype that is associated with the shortest survival and the highest risk of AML transformation (mean expression value of GLS was 493 in controls vs 658 in RAEB, *p* = 0.01) (Fig. 1A). We also observed that patients with higher expression of GLS had a significantly worse survival (median overall survival of 2.7yrs in high GLS vs 5.7yrs in low GLS expressors, log rank *p* = 0.003) (Fig. 1B).

GLS1 is present in two isoforms; a longer isoform called kidney glutaminase (KGA); and a shorter isoform called glutaminase C (GAC), which lacks the C-terminal region. GAC isoform was found to be more active and frequently upregulated in diverse types of cancers ^22^. Immunoblotting demonstrated that GAC isoform was present in numerous AML cell lines and was more prevalent than KGA isoform (Fig. 1C). GAC showed marked upregulation under hypoxic conditions resembling leukemia bone marrow microenvironment^23^ (lane 3), or in cells overexpressing HIF-1α (Fig. 1D).

### Silencing or pharmacological inhibition of GLS reduces cell growth and is synergistic with hypomethylating agent azacytidine

To determine the functional role of GAC and KGA isoforms in leukemic cells, we generated stable knockdown with doxycycline inducible shRNA of the GAC, KGA or GAC/KGA isoforms of GLS ^24^ in MV4-11 and OCI-AML3 cells (Fig. 1E). Doxycycline-inducible GAC and GAC/KGA knockdown resulted in reduction in intracellular Glu and Asp, a corresponding increase in Gln (Fig. 1F) and reduction of oxygen consumption (OCR) (**Supplementary Fig. 1C**), resulting in cellular growth inhibition, while metabolic changes upon KGA knock-down were less profound and did not affect cellular growth (Fig. 1G).

Consistently, GLS inhibition by CB-839 reproduced OCR inhibition, as shown upon GLS KO or GLS inhibition with BPTES (**Supplementary Fig. 1D-E**) and was accompanied by an increase of glutamine, indicating an on-target effect, with a statistically significant decrease of glutamate, aspartate, glutathione. Furthermore, a significant reduction of all tricarboxylic acid cycle (TCA) intermediates was observed (Fig. 1H). Gln has been shown to play a role in the generation of oncometabolite 2-hydroxyglutarate (2-HG) in tumors with high GLS expression, whereby glutamine-derived α-ketoglutarate is reductively carboxylated by IDH2 enzyme to form isocitrate and 2-HG^25,26^. We demonstrated that HL60 cell line subjected to GLS inhibition showed a reduced 2-HG level (Fig. 1H). 2HG in turn is known also to inhibit 2-oxoglutaratre-dependent dioxygenases such as TET enzymes leading to decreased hydroxymethylation. ^27^ Consistently, treatment of leukemia cells with CB-839 caused statistically significant reduction of monophosphatenucleotides: AMP and UMP an increase in cytosine hydroxymethylation (Fig. 1I and **Supplementary Fig. 1F**).

### Anti-leukemia efficacy of combination of GLS inhibition and DNMT inhibition in vitro and in vivo

**Figure 2:**
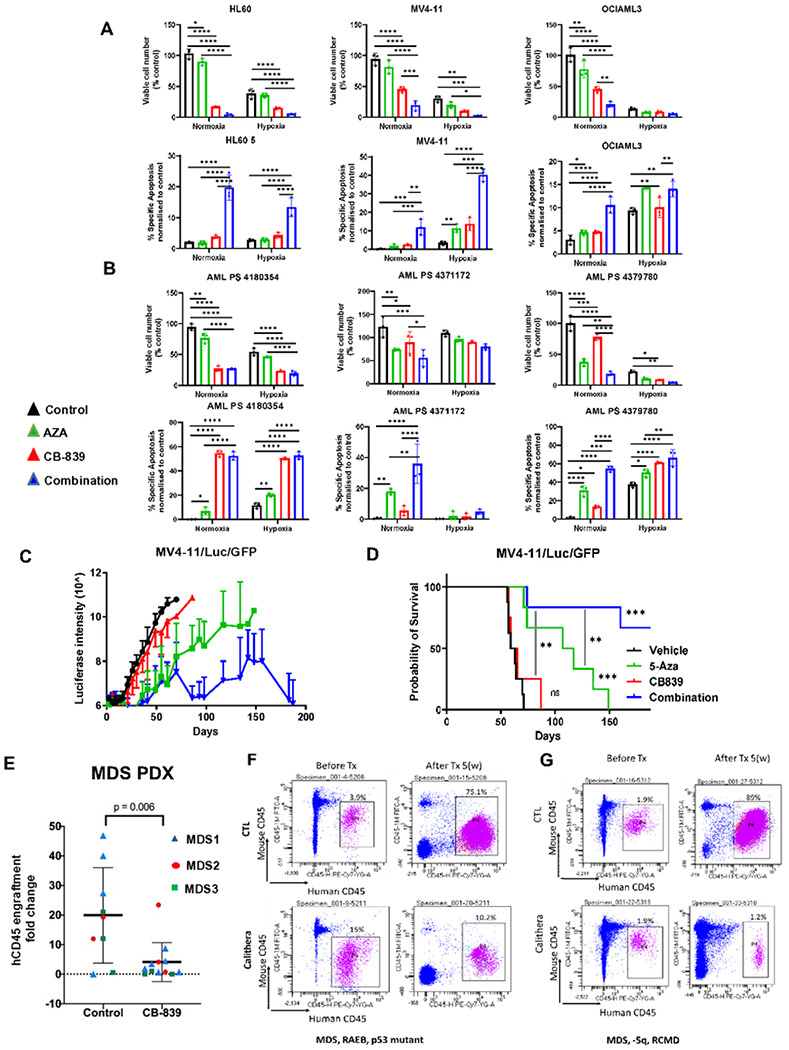
Anti-leukemia efficacy of AZA/CB-839 in AML cell line, primary AML cells and MDS patients. **(A, B)** Treatment with 1 μM of telaglenastat (CB-839) and escalating doses of AZA (HL-60 2.5 μM, OCIAML3 and MV4-11 1 μM) caused additive or synergistic inhibition of cellular growth after 3 or 5 days of culture, both under normoxia and hypoxia, in AML cell lines (OCI-AML3, HL-60, MV4-11) **(A)** and primary AML cells (n=3) **(B).** Viable cell number are presented in top panel, and apoptosis measurement in bottom panels, (t-test, n=3 replicates) Significance was determined using unpaired two-tailed t-test annotated as * p ≤ 0.05, ** p ≤ 0.01 *** p = 0.001, **** p ≤ 0.001. **(C, D)** MV4-11/Luc/GFP cells (1×10^6^ per mouse) were injected intravenously into NSG-S mice. Starting on day 6, mice (n = 6 mice per group) were treated with a vehicle, 5 mg/kg AZA, 200 mg/kg telaglenastat (CB-839), or both. AZA was administered IP, qd for 5 days, and CB-839 po, q12h for 6 weeks. The luciferase intensity was quantified by serial bioluminescence imaging from 6 representative mice from 4 groups **(C).** Overall survival in each group of mice was estimated by the Kaplan-Meier method **(D).** **(E)** 3 different MDS patient-derived xenografts were prepared by transplantation of peripheral blood or BM mononuclear cells (MNCs) into irradiated NSG mice. After confirmation of engraftment by serial BM samples, the cohort was split and treated with either drug at a dose of 200 mg/kg or control for 5 weeks. Human CD45^+^ engraftment was calculated, and fold change of Post/Pre-treatment engraftment was shown for Ctrl and telaglenastat (CB-839) treated mice. (*p*=0.037) **(F-G)** MDS samples from low and high-risk patients analyzed after 5 weeks of treatment shows increasing engraftment in the control-treated mice but a loss of graft in CB-839 treated mice, as demonstrated by the human CD45 antibody.

Given the previously described potency of telaglenastat (CB-839) to induce hydroxymethylation, we next examined the potential synergy of CB-839 with the DNMT3A inhibitor azacytidine (AZA) in leukemic models. Treatment with 1 μM CB-839 and escalating doses of AZA caused synergistic inhibition of cellular growth after 5 days of culture, both under normoxia and hypoxia, in cell lines as well as in primary AML cells (n = 3) (Fig. 2A,B), significantly decreasing viable cell number and increasing apoptosis, seen in combination (Fig. 2A,B).

To test the efficacy of both compounds *in vivo*, we injected NSG-S mice with genetically engineered MV4-11/Luc cells. Consistent with our *in vitro* data, in MV4-11/Luc cell line-derived xenograft model, cotreatment with telaglenastat and AZA significantly reduced leukemia burden in treated groups compared to controls as demonstrated by bioluminescent imaging (BLI) (Fig. 2C) and resulted in significant extension of survival when compared with both vehicle and AZA single agent, *p* < *0.001* (Fig. 2D).

We wanted next to determine the *in vivo* efficacy of telaglenastat, against primary MDS xenografts. Peripheral blood– or BM–derived mononuclear cells (MNCs) from MDS patients were transplanted into irradiated NSG mice. After confirmation of engraftment by serial BM samples, the cohort was split and treated with either CB-839 at a dose of 50 mg/kg or vehicle control for 5 weeks. Telaglenastat treatment led to decreased MDS burden in xenografted mice as compared with control (Fig. 2E). This was seen in both low-risk MDS (Fig. 2F) and high-risk MDS models (Fig. 2G), supporting activity against human xenografted cells *in vivo*.

### Clinical efficacy of combination of Telaglenastat and azacytidine in MDS

Based on the efficacy of combined inhibition of GLS and DNMT3A in pre-clinical studies, a phase Ib/II study of telaglenastat in combination with AZA in patients with higher risk myelodysplastic syndrome was initiated. 30 patients with myelodysplastic syndromes were enrolled; 2 patients were screen failures and 28 of 30 enrolled patients received treatment (**Supplementary Fig. 2**). The baseline characteristics of the 28 treated patients are provided in **Supplementary Table 1.** The median age was 70 years (range 41–83 yrs). Ten patients (36%) had received prior MDS-directed therapy, including 9 with prior HMA exposure (median 6 prior cycles, range 4–9) and one non-HMA based regimen. Nine (32%) patients had a diagnosis of therapy related MDS, and seven (25%) had CMML. Cytogenetics and mutational status were evaluated locally prior to treatment initiation. Ten (36%) patients had complex cytogenetics. The most frequent mutations at study enrollment were *ASXL1* (n = 14; 50%), *TET2* (n = 11, 39%), *TP53*(n = 10; 36%) and *RUNX1* (n = 9; 29%).

All patients received standard azacytidine 75 mg/m2 daily x 7 days by intravenous or subcutaneous injection, and telaglenastat was self-administered orally twice daily continuously, for 28 days per cycle. As of data cutoff (October 10, 2022), of the 28 treated patients, 7 are alive, and none remain on active treatment. Patients discontinued treatment for the following reasons: loss of response or progression (n = 11), the decision to proceed with stem cell transplant (n = 7), death on study for reasons not related to study therapy (n = 7), patient decision (n = 2), and continued protocol non-compliance (n = 1). Five patients achieved a complete remission (CR) (18%), and 10 patients (36%) achieved a marrow CR (mCR), including 4 patients with mCR and hematologic improvement (platelet improvement in 2 patients; platelet and neutrophil improvement in 1 patient, and platelet and hemoglobin improvement in 1 patient) leading to an overall response rate of 54%. Three additional patients experienced a hematologic improvement in platelets, hemoglobin, or neutrophil counts, respectively. Ten patients had no response or a best response of stable disease.

**Figure 3:**
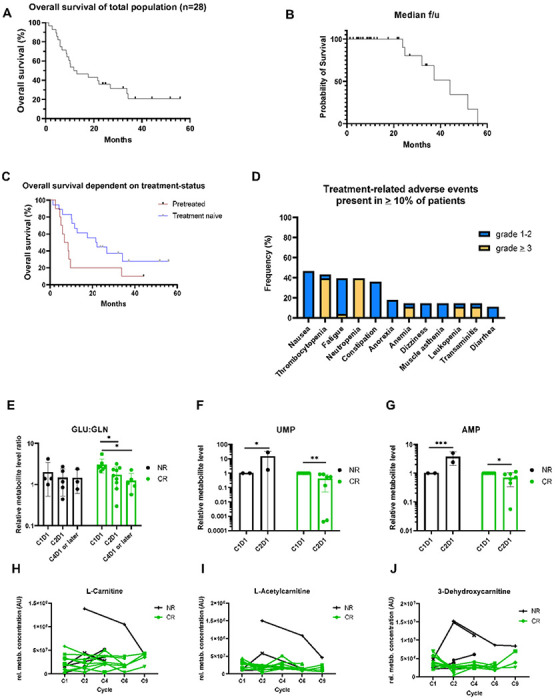
Telaglenastat demonstrates on-target efficacy linked to modulation of intracellular metabolism. **(A)** Overall survival of all n=28 enrolled into clinical trial patients. **(B)** Median f/u in the study. **(C)** Comparison of overall survival of all n=28 in reference to treatment status **(D)** Frequency of treatment related adverse events presented in ≥ 10% of patients. **(E)** Ratio of the intracellular levels of glutamate and glutamine dropped in most patients, consistent with the know mechanism of telaglenastat. The peripheral blood cells were collected from patients before treatment, at the end of cycle 1, 2 and 4. The cells were collected for metabolic analysis. **(F)** UMP level among responders and non-responders, and **(G)** AMP concurrently showed consistent trends associated with patient response early during treatment. **(H-J)** Mass spectrometry analysis of Carnitine, Acetylcarnitine and Dehydroxycarnitine respectively in reference to treatment response. **(H)** Any Correlation?

Time to response occurred at a median of 2.3 cycles (range 1 –4). With a median follow-up time of 30.9 months, median overall survival (OS) was 12.3 months with a 1-yr OS of 50%. In treatment naïve patients, median OS (mOS) was 22.2 months and in previously treated patients, the mOS was 7.7 months. Full complete remissions (CR) were exclusively observed in treatment-naïve patients: of the 18 treatment-naïve patients, 5 had CR, 5 had mCR, and 1 had hematologic improvement (HI); for an overall response rate of 61% (11/18). Of the 10 previously treated patients, 7 experienced a mCR (n = 5) or HI (n = 2) for an ORR of 70%. Of the 10 patients with complex cytogenetics, there were 7 responses including 2 CRs, 4 mCR and 1 HI. Of the 7 patients with CMML, there were 2 CR and 2 mCR. Additional details are provided in Fig. 3, Table 1, **Supplementary Tables 2.**

With a median follow-up time of 44.2 months, median overall survival was 11.6 months with a 1-yr OS of 46.4% (Fig. 3A-B, **Supplementary Fig. 3A-C**). In treatment naïve patients, median OS (mOS) was 21.6 months, while. patients, of which 9 of 10 previously received AZA or DAC, had mOS of 6.8 months (Fig. 3C).

Plasma levels of telaglenastat achieved in patients and quantified by LC-MS did not show statistically significant correlation with clinical response, however patients in CR/mCR group demonstrated a higher level of CB-839 in plasma than patients in HI/NR group. Of note, at the start of cycle 3, patients in CR/mCR group reached an average stable level of CB-839 of over 1,055 (365-2,534) ng/ml, while patients in HI/NR group had a wider range of telaglenastat in serum with 575 (0–2,879) ng/ml (**Supplementary Fig. 3D-E).**

### Safety of combination of Telaglenastat and Azacytidine in MDS:

The combination of azacytidine and telaglenastat was generally well tolerated. The most common non-hematologic adverse events (AEs) of any grade, regardless of attribution, were nausea (52%), constipation (39%), anorexia (17%), and generalized weakness (17%). The most common grade ≥ 3 AEs was transaminitis (13%), along with the hematologic adverse events of neutropenia (30%) and thrombocytopenia (26%). Seven patients received dose reductions of telaglenastat on study, due to transaminitis (n = 2), myalgias and fatigue (n = 2), and prolonged cytopenia in the setting of marrow CR (n = 3). No cases of tumor lysis syndrome or differentiation syndrome were reported. 30 and 60-day mortality were 0% and 3.6%, respectively (Fig. 3D).

### Target engagement and identification of putative metabolic markers of response

Consistent with the known mechanism of GLS inhibition, telaglenastat caused a decrease in the ratio of intracellular glutamate to glutamine (GLU/GLN) levels in peripheral blood cells of patients that responded to telaglenastat (Fig. 3E).

Other potential metabolic markers of early response to treatment were evaluated in the patient’s peripheral blood cells. Uridine monophosphate (UMP) and adenosine monophosphate (AMP) intracellular levels markedly increased in patients that did not respond to treatment, and moderately decreased in patients that responded (Fig. 3F,G).

Also, early during treatment, the intracellular levels of several carnitine metabolites decreased in most patients that responded to treatment (Fig. 3H-J), while increasing or remaining elevated in non-responders. These results indicate metabolic rewiring towards fatty acids β-oxidation and a reduced propensity of MDS cells to compensate for the blockade of glutamine metabolism via activation of fatty acids metabolism as a feature/biomarker of response to telaglenastat.

### Responders’ bone marrows demonstrate stem cell differentiation program at single cell level

**Figure 4:**
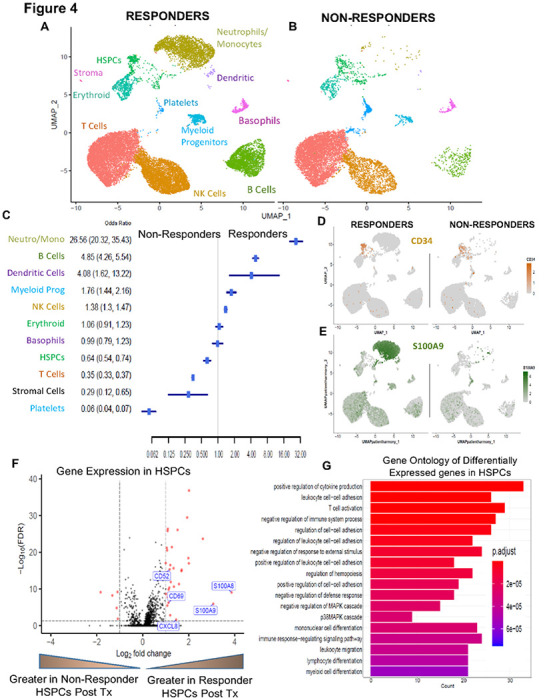
Glutaminase inhibitor treated MDS Bone marrows demonstrate stem cell differentiation at single cell level in clinical responders **(A-B)** scRNAseq was conducted on bone marrow cells from telaglenastat treated patients. 5 clinical responders and 3 non responders were analyzed, and 11 distinct cell populations identified based on gene expression patterns. **(C)** Cell populations that were significantly enriched in responders and non-responders are shown (Odd’s ratio (OR) with 95% CI, Fisher’s Test). Significantly increased numbers of Neutrophil / Monocytic cells were seen in post treatment responder samples. **(D)** CD34 expressing HSPC population was enriched in non-responders and are shown in representative UMAP figures. **(E)** S100A9 expressing Neutrophil/Monocyte population was enriched in responders and are shown in UMAP figures. **(F)** Volcano plot of differentially expressed genes in HSPCs shows responders with enrichment in severa myeloid differentiation associated genes **(G)** Gene ontology of differentially expressed genes in HSPC demonstrates enrichment for leucocyte related genetic programs.

To further evaluate the response to telaglenastat and AZA combination at the single cell level, we performed scRNAseq on post treatment bone marrows of 4 responders and 3 non-responder patients with enough viable cells. A total of 38,195 cells were analyzed and 11 distinct cell populations were identified based on gene expression patterns (Fig. 4A,B). We observed that cell populations that were enriched in responders included neutrophil/monocyte and myeloid progenitor cell types (Fig. 4C). These were validated by expression of stem cell markers (CD34) and differentiation markers (S100A8,9, CXCL8) in the respective cellular clusters (Fig. 4D,E, **Supplementary Fig. 4**). Analysis of differentially expressed genes within the discrete HSPC population revealed increased expression of myeloid differentiation genes in the responders (Fig. 4F). In fact, the responder HSPC transcriptomes revealed enrichment for leukocyte/myeloid functional pathways (Fig. 4G). These results demonstrate that responders to the drug combination have significant enrichment for myeloid differentiation programs in stem cells and suggest that this therapy relieves the MDS differentiation blocks *in vivo*.

Reduction in Leukemic Stem Cells and increased stem cell differentiation is seen in responders to azacytidine/Telaglenastat

**Figure 5:**
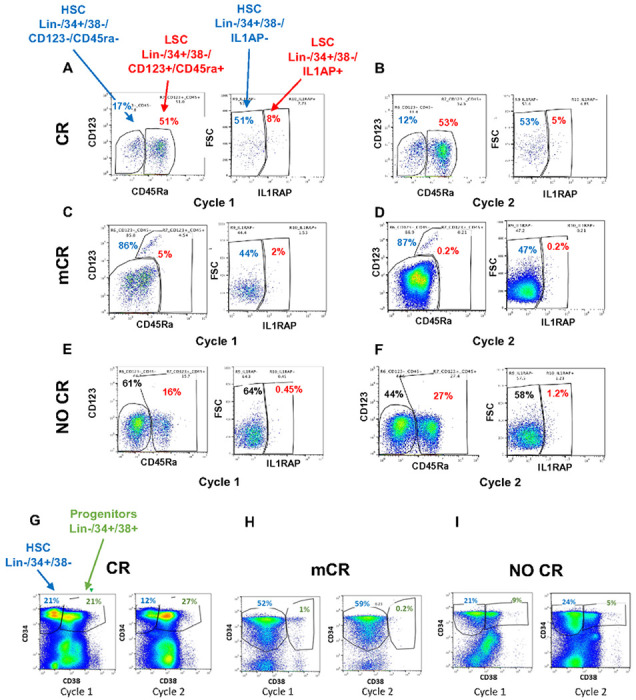
Reduction in Leukemic Stem Cells and increased differentiation is seen in responders to AZA/Telaglenastat combination **(A-D)** Flow done on serial bone marrows from patients in CR **(A, B)** and marrow CR **(C, D)** showed reduction in LSCs (CD34^+^/CD38^−^Lin^−^ve with IL1RAP^+^ or CD34^+^/CD38^−^/Lin^−^ve with high CD45^+^/CD123^+^). **(E, F)** BM from patient with no response showed no reduction in IL1RAP^+^ or CD45/CD123 high LSCs in serial bone marrows. **(G-I)** Bone marrow from patient in CR showed HSC (CD34^+^/CD38^−^) to progenitor (C34^+^/CD38^+^) differentiation in serial BM **(G)**. Patient with mCR without count recovery and non-responding patients did not show differentiation **(H)**. Myeloid differentiation at the stem cell level is seen in responding patients**(I)**.

Since leukemic stem cells have been shown to contribute to treatment failures and are not eliminated by azacytidine^28^, we determined their dynamics after treatment with AZA/telaglenastat combination. We performed multicolor flow cytometry on serial bone marrows to assess stem and progenitor cell numbers from 3 representative patients that achieved a full CR, a marrow CR and no response. Assessment of normal and leukemia stem cells (LSCs) was conducted using schema published previously ^29^ with a gain of IL1RAP positivity or high CD123 positivity indicative of leukemic stem cell phenotype. Flow cytometry performed on serial BM from patients in CR (Fig. 5A,B) and marrow CR (Fig. 5C,D) showed reduction in IL1 RAP + LSCs (CD34^+^/CD38^−^/Lin^−^ve with IL1RAP^+^) or CD123 + LSC (CD34^+^/CD38^−^/Lin^−^ve with high CD45^+^/CD123^+^) in the two cases respectively. BM from a patient with no hematologic or marrow blast response showed no reduction in IL1RAP^+^ or CD123 high LSCs in serial bone marrows (Fig. 5E,F).

We also assessed for differentiation at the stem cell level in these representative cases. BM from patient in CR showed HSC (CD34^+^/CD38^−^) to progenitor (C34^+^/CD38^+^) differentiation in serial bone marrows (Fig. 5G). Patient with mCR without count recovery and non-responding patients did not show differentiation (Fig. 5H,I), consistent with no peripheral blood count recovery.

Glutamine transporter, SLC38A1, is overexpressed in MDS/AML stem cells; is associated with an adverse prognosis and correlates with response to AZA/Telaglenastat

**Figure 6:**
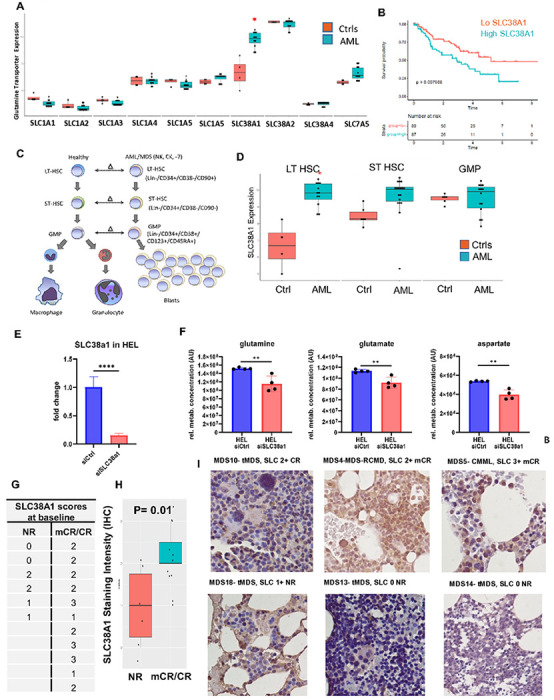
Glutamine transporter, SLC38A1, is overexpressed in MDS/AML stem cells; is associated with an adverse prognosis and correlates with response to response to AZA/Telaglenastat **(A)** Expression of all known glutamine transporters in LT HSC (Lin^−^ve, CD34^+^, CD38^+^, CD90) from controls and AML were evaluated and SLC38A1 was the only transporter that was significantly overexpressed in AML LT-HSCs. SLC38A1 was most significantly overexpressed in AML LT HSCs and ST HSCs with −7 and Complex karyotypes. **(B)** Survival of 183 MDS patients was correlated with SLC38A1 expression in marrow derived CD34^+^ cells. Patients with higher SLC38A1 levels (greater than median) had a median survival of 2.6 years compared with 5.8 years for the group with lower STAT3 (log-rank *p < 0.01*). **(C)** Schematic figure showing stem cell differentiation stages, at which cells were harvested and analyzed. To determine glutamine transporter expression levels in highly purified AML/MDS stem and progenitor cells, we examined gene expression profiles generated from FACS-sorted LT-HSCs, ST-HSCs, and GMPs from 12 MDS/AML samples with normal karyotype, deletion of chromosome 7, and complex karyotype (Gene Expression Omnibus [GEO], GSE35008 and GSE35010). **(D)** Gene expression levels in highly purified AML/MDS stem and progenitor cells obtained from FACS-sorted LT-HSCs, ST-HSCs, and GMPs from 12 MDS/AML samples with normal karyotype, deletion of chromosome 7, and complex karyotype (Gene Expression Omnibus [GEO], GSE35008 and GSE35010) as described in **(A)**. **(E)** Evidence of successful knockdown of SLC38A1 with siRNAs measured by RT-PCR. **(F)** The intracellular levels of glutamine, glutamate and aspartate decrease in leukemic HEL cells following knockdown of the glutamate transporter SLC38A1 with siRNA. Knockdown of SLC38A1 led to reduced glutamine transport in leukemic HEL cells as measured by LMSC, (mean ± SD, n=4 replicates per condition); (t-test, *p<0.05*). **(G-I)** Baseline BM biopsy sections from 17 patients with MDS that were treated with telaglenastat (CB-839) and azacytidine (AZA) were immunohstochemically stained with antibody against SLC38A1. Specific membrane/cytoplasmic staining was seen in BM cells and graded based on intensity as shown in representative sections. Patients with response (marrow CR/CR) to Glutaminase inhibitor/AZA treatment demonstrated a higher baseline mean SLC38A! Expression (t-test, *p=0.01*)

Glutamine can be transported inside cells via various transporters, and we wanted to determine whether their expression would predict responsiveness to telaglenastat treatment in MDS. First, to determine glutamine transporter expression levels in highly purified AML/MDS stem and progenitor cells, we examined gene expression profiles generated from FACS-sorted LT-HSCs, ST-HSCs, and GMPs from 12 MDS/AML samples with normal karyotype, deletion of chromosome 7, and complex karyotype (Gene Expression Omnibus [GEO], GSE35008 and GSE35010). Expression of all known glutamine transporters including canonical SLC1A1-5 and non-canonical SLC38A1-3 were evaluated and SLC38A1 was the only transporter that was found to be significantly overexpressed in AML LT-HSCs (Fig. 6A). Next, to determine the prognostic impact of SLC38A1 expression, we correlated the survival of 183 MDS patients with SLC38A1 expression in marrow derived CD34^+^ cells. Patients with higher SLC38A1 levels (greater than median) had a median survival of 2.6 years compared with 5.8 years for the group with lower SLC38A1 (log-rank p < 0.01) (Fig. 6B). Subtype analysis showed that SLC38A1 was most significantly overexpressed in AML LT-HSCs and ST-HSCs with the worst prognosis in patients with poor risk monosomy 7 and complex karyotypes (Fig. 6C-D).

To determine the functional relevance of this transporter in leukemic cells, we used siRNAs for a specific knockdown in leukemic cells (Fig. 6E). Knockdown of SLC38A1 with siRNAs led to significantly reduced glutamine transport in leukemic HEL cells as measured by metabolic flux experiment (Fig. 6F). Lastly, we obtained baseline BM biopsy sections from 17 patients with MDS that were treated with telaglenastat and azacytidine in our trial and performed immunohistochemical staining with antibody against SLC38A1. Specific membrane/cytoplasmic staining was seen in BM cells and graded based on intensity as shown in representative sections (Fig. 6G-I). Patients with response (marrow CR/CR) to AZA/telaglenastat treatment demonstrated a significantly higher baseline mean SLC38A1 expression.

## Discussion

The combination of standard of care azacytidine and the glutaminase inhibitor telaglenastat was well tolerated in MDS and showed target inhibition *in vivo*. A 54% objective response rate by IWG criteria was observed, with an additional 11% of patients experiencing unilineage hematologic improvement and 25% of patients successfully transitioning to allogeneic stem cell transplantation. Activity was observed in high-risk MDS patient subsets, including those with previously treated (i.e. the poor prognosis “HMA-failure” population), those with complex cytogenetics and/or TP53 mutations, and patients with CMML.

Analysis of our unique dataset of purified MDS stem/progenitor cells demonstrated upregulation of GLS specifically in stem cells of high-risk MDS and an association with inferior prognosis. These findings point to key role of glutaminase in MDS stem cells. These results were supported by the demonstration of *in vivo* activity against LSCs with AZA +telaglenastat treatment, which has not been observed in the setting of AZA treatment alone suggesting telaglenastat combinations may offer future LSC-directed therapy. Additionally, the increase in myeloid progenitor cells seen in responding patients is consistent with myeloid differentiation.

Myeloid differentiation was further demonstrated in single cell RNAseq performed on post treatment samples. The clinical responders had a significant increase in myeloid differentiation program in their stem and progenitor cells. Also, we observed an increased population of differentiated myeloid cells in responder bone marrows. Stem and progenitor differentiation blocks are a hallmark of MDS and lead to cytopenias as the dominant clinical manifestation in these diseases. Our data provide the first evidence of *in vivo* differentiation after treatment with an epigenetic and metabolic drug combination in MDS.

Recent reports indicate overexpression of specific glutamine transporters in cancers ^30,31^. Our gene expression analysis of various glutamine transporters demonstrated selective overexpression of SLC38A1 in LT-HSCs in patients with MDS and AML. Patients with poor risk complex karyotype and monosomy 7 have the highest SLC38A1 expression, and high SLC38A1 is associated with decreased survival. Mechanistically, silencing of SLC38A1 translated into inhibition of intracellular glutamine and its downstream metabolites glutamate and aspartate, supporting the functional role of this transporter in leukemic cells. Importantly, elevated protein expression of SLC38A1 was observed in BM from patients who responded to telaglenastat and azacytidine therapy, implicating SLC38A1 as biomarker of response to treatment.

In summary, our findings support the previously unrecognized role of glutamine in stem cells of high risk MDS patients, associated with overexpression of the specific glutamine transporter SLC38A1 facilitating Gln uptake and its utilization, such as nucleotide biosynthesis. Our findings indicate that activation of this metabolic branch is associated with high-risk disease and inferior outcomes, supporting further exploration of GLS inhibition in patients with myeloid malignancies. Alternatively, targeting SLC38A1 or other glutamine-related enzymes may offer novel therapy in MDS/AML, with recent examples of targeting glutaminolysis ^32–35^. These results support the ongoing exploration of GLS inhibition with telaglenastat combinations in patients with myeloid malignancies.

## Methods

### Primary AML and MDS blast cells

Primary AML and MDS cells were collected from patients who had consented to research protocols approved by the Institutional Review Board at The University of Texas MD Anderson Cancer Center for analysis of hematologic malignancies. BM aspirate mononuclear cells from patients with MDS were purified by Ficoll density centrifugation using standard procedures. Lymphocytes were isolated by using Lymphocyte Separation Medium (SIGMA).

Primary AML peripheral blood mononuclear cells (PBMCs) were cultured in serum-free Expansion Medium (Cat. 09650) supplemented with BIT 9500 Serum Substitute (Cat. 09500; both from STEMCELL Technologies Inc., Vancouver, BC, Canada) and cytokines including stem cell factor (SCF, 100 ng/mL, Cat. 300-07), Flt3 ligand (50 ng/mL, Cat. 300 – 19), IL3 (20 ng/mL, Cat. 200-03), and G-CSF (20 ng/mL, Cat. 300 – 23; all from Peprotech, Rocky Hill, NJ) and StemRegenin 1 (1 μM, Cat. S2858; Selleck Chemicals LLC, Houston, TX).

#### Apoptosis And Cell Growth Of AML Cells And Primary AML Samples

AML cell lines and primary AML cells were subjected to treatment with telaglenastat (CB-839) and/or azacytidine (AZA) for 3 or 5 days. Cells were then washed in Annexin-V binding buffer and stained with a cocktail of antibodies comprising CD45-FITC (Cat. 347463) and Annexin-V APC (Cat. 550475; both from BD Biosciences, San Jose, CA) for 30 minutes at room temperature in the dark. The cells were then washed and resuspended in Annexin-V binding buffer with 4 6-diamidino-2-phenylindole (DAPI). Viable AML cells were enumerated by using CountBright counting beads (Cat. C36950; Invitrogen, Carlsbad, CA) with concurrent Annexin-V and DAPI detection on a Gallios Flow Cytometer (Beckman Coulter, Indianapolis, IN). Data were analyzed by using Flowjo software (Tree Star, Ashland, OR).

#### Animal Xenograft Models

All animal studies were performed under protocols approved by the institutional animal care and use committee (IACUC) at M.D. Anderson Cancer Center and Albert Einstein College of Medicine. For AML cell line derived xenograft studies, 6–8-week-old female NSGS mice (NOD-scid IL2Rgnull-3/GM/SF, NSG-SGM3, (RRID:IMSR_JAX: 013062); mice were obtained from Jackson Laboratory, Bar Harbor, ME, housed, and handled in the animal facility of MD Anderson Cancer Center. To determine the *in vivo* effects of telaglenastat (CB-839) and/or azacytidine (AZA) on leukemia progression and engraftment, 1 million MV4-11/Luc/GFP cells were injected into the lateral tail vein of NSGS mice, which had received a preconditioning dose of radiation (2.5 Gy) 24 h prior to injection of cells. Starting on day 6, mice (n = 6 per group) were treated with a vehicle, 5 mg/kg AZA, 200 mg/kg CB-839, or both. AZA was administered ip daily for 5 days, and CB-839 po twice a day for 6 weeks. The luciferase intensity was quantified by serial bioluminescence imaging from 6 representative mice from 4 groups. The survival of the mice is represented by a Kaplan-Meier plot.

For primary patient derived MDS xenografts NOD/SCID IL2Rgamma KO NSG mice were bred, housed, and handled in the animal facility of Albert Einstein College of Medicine. NSG mice were irradiated (2 Gy) 24 hr prior to injection. Mononuclear cells from primary MDS patients were isolated by Ficoll separation. 2–5 × 10^6^ MNCs were administered via tail vein injection.

3–4 weeks later, BM aspiration were performed and analyzed by flow cytometry for the human cell engraftment. Mice were considered to be engrafted if they showed 0.1% or higher human derived CD45^+^ cells. The engrafted mice were randomized for treatment with 200 mg/kg mg/kg/d of telaglenastat or control for indicated times. Following treatment, BM aspirations and flow cytometry analysis for the above-mentioned markers were performed. All mice were bred, housed, and handled in the animal facility of Albert Einstein College of Medicine.

#### Patients

Eligible patients had a confirmed diagnosis of MDS, classified as Int-2 or high-risk MDS by the IPSS, or Int-1 risk MDS with the presence of high-risk mutations (i.e. *ASXL1, TP53, RUNX1, EZH2*).^36,37^ Eligibility included adult patients with an Eastern Cooperative Oncology Group Performance score 0–2; adequate cardiac, renal and liver function, and without active infection or CNS disease. Patients with clinically significant gastrointestinal conditions that could interfere with study drug absorption were excluded. Receipt of prior MDS-directed therapies was allowed.

This study was approved by the MD Anderson Cancer Center Institutional Review Board and was conducted in accordance with the Declaration of Helsinki. Informed consent was obtained from all participants.

#### Study Design And Analysis

This Phase 1b/2 clinical trial (NCT03047993) was designed to assess the safety, tolerability, and efficacy of telaglenastat in combination with azacytidine for the treatment of advanced MDS.

The study was fully completed with twenty-eight patients enrolled from December 2017 to September 2020: six in the Phase 1b portion and twenty-two in the Phase 2. All patients received standard azacytidine 75 mg/m2 daily x 7 days by intravenous or subcutaneous injection, and telaglenastat was self-administered orally twice daily continuously, for 28 days per cycle.

The Phase 1b study portion was designed to enroll six patients, treated at the standard approved azacytidine dose, in combination with telaglenastat at a dose of 600mg BID. If ≤ 1 DLT was observed at this combination, this level would be identified as the recommended phase 2 combination dose (RP2D) and the study would progress to the Phase 2 portion. If ≥ 2 patients experienced a DLT, an additional six patients would be enrolled at the next lower dose level. The Phase 1b portion enrolled without reported DLTs and the study moved into the Phase 2 portion.

The Phase 2 portion opened after confirmation of the combination RP2D of 600mg BID. One interim analysis was designed after 16 patients enrolled, with a plan to continue total accrual if ≥ 7 of the first 16 patients achieved response. The final efficacy analysis was designed such that if 15 or more of the 28 enrolled patients achieved response, treatment would be considered efficacious and worthy of further investigation.

Clinical response was evaluated using the modified IWG response criteria for MDS.^38^ The overall response rate included complete remission (CR), partial remission (PR), and marrow CR (mCR). Hematologic improvement lasting ≥ 8 weeks was also assessed. BM response assessments occurred after completion of cycle 1, cycle 3, cycle 5, and every 3 cycles thereafter. Overall Survival (OS) and Progression-Free Survival (PFS) was calculated by Kaplan-Meier method. Safety was assessed in all patients; AEs were graded according to the Common Terminology Criteria for Adverse Events (CTCAE) v 4.0.

Treatment continued until discontinuation due to disease relapse or progression, lack of clinical benefit, unacceptable toxicity, transition to allogeneic stem cell transplant, or patient decision. Dose interruption or dose reductions were permitted if toxicities were observed. Supportive care therapy was allowed as per institutional standards.

#### Correlative Biomarkers

PK studies were performed on plasma on C1D15, C2D1, and C3D1 to assess steady-state levels of telaglenastat over time, and drug accumulation over time. Pharmacodynamic (PD) effects of telaglenastat were measured in CD34^+^ and BM stromal cells isolated before treatment and at all BM study timepoints. Measurements of intracellular metabolites, RNA expression of GLS and other metabolic genes, and quantitation of phenotypically defined MDS stem cells were planned as exploratory analyses.

#### Metabolomics Analysis

Intracellular metabolites were extracted using a modified Bligh-Dyer procedure and analyzed by an ultra-high pressure liquid chromatography Vanquish tandem Q-Exactive mass spectrometer system (Thermo Scientific, Waltham, MA), as previously described^39,40^. The MS was calibrated for mass accuracy before analysis and monitored throughout data acquisition to maintain mass accuracy below 5 ppm. A pooled quality control sample was injected between every batch of 6 samples to monitor instrument performance and guarantee consistency across the runs. Initial chromatographic separation of polar metabolites was performed using an Atlantis Premier BEH Z-HILIC Column, 1.7 μm, 2.1 mm X 150 mm (Waters). The mobile phases used for this analysis were A) LCMS-grade water + 10 mM ammonium acetate, B) 90:10 acetonitrile:LCMS-grade water + 10 mM ammonium acetate (pH of 9.2), C) 100% acetonitrile. The following parameters were used for separation: sample injection volume: 5 μL; flow rate: 0.15 mL/minutes; flow gradient: 90:10 A:C for 7 minutes, 100% B for 10 minutes, 90:10 A:C for 18 minutes. After initial data acquisition, a CentriVap Benchtop Concentrator (Labconco) was used to dry the samples before they were resuspended in LCMS-grade water for secondary analysis using a Kinetex C18 Column, 2.6 μm, 100 Å, 150 × 2.1 mm (Phenomenex). The mobile phases for this analysis were A) LCMS-grade water + 0.2% formic acid, B) 100% methanol. The following parameters were used for separation: sample injection volume: 5 μL; flow rate: 0.15 mL/minutes; flow gradient: 98:2 A:B for 4 minutes, 20:80 A:B for 10 minutes, 2:98 A:B for 7 minutes, 98:2 A:B for 14 minutes. The raw data files were processed with SIEVE 2.2.0 software (Thermo) and the integrated peaks were mined against an in-house database of standards that includes the IROA Mass Spectrometry Metabolite Library of Standards (MSMLS; IROA Technologies) for accurate masses and retention times. The peaks were also matched to the accurate masses of the Human Metabolome Database^41^.

#### Single Cell RNA-Seq and paired B-cell or T-cell receptor (BCR/TCR) sequencing

scRNA-seq libraries were generated using a 10x Genomics Chromium Controller instrument and Chromium Single-Cell 5’ V2 reagent kits (10x Genomics). In brief, cells were concentrated to 1,000 cells per μl and cells were loaded into each channel to generate single-cell Gel Bead-in-Emulsions (GEMs), resulting in mRNA barcoding of an expected 5,000 single cells for each sample. After the reverse transcription step, GEMs were broken, and single-strand cDNA was cleaned up with DynaBeads. The amplified barcoded cDNA was fragmented, A-tailed, ligated with adaptors and amplified by index PCR. cDNA and constructed libraries were assessed by High Sensitivity D5000 DNA Screen Tape analysis (Agilent Technologies) and Qubit dsDNA HS Assay Kit (Thermo Fisher Scientific). Sequencing was conducted on an illumina NovaSeq sequencer with 2 × 100 bp paired reads to reach a depth of at least 50,000 read pairs per cell. The Chromium Single-Cell V(D)J Enrichment Kit was used to enrich immune repertoire, TCR or B-cell immunoglobulin (Ig) transcripts. The single-cell V(D)J enriched libraries were sequenced at 2 × 150 bp with a minimum depth of 5,000 read pairs per cell.

#### scRNA-Seq Bioinformatics Analysis

Raw scRNA-seq data was pre-processed, demultiplexed, and aligned to human reference genome (GRCh38) using CellRanger (10X Genomics). Cells with few (< 200) or many (> 6,000) genes, likely doublets or multiplets predicted by Scrublet ^42^, and cells with > 25% of read counts derived from the mitochondrial genome were removed. The batch effects were corrected by Harmony ^43^. Raw unique molecular identifier (UMI) counts were log-normalized ^44^ and used for principal component analysis (PCA). Seurat v4^44^ was applied to the normalized gene-cell matrix to identify highly variable genes for unsupervised cell clustering. Both Uniform Manifold Approximation and Projection (UMAP) ^45^ method and the tSNE method were used for dimensionality reduction and two-dimensional visualization of the single-cell clusters.

To define the major cell type and state of each single cell, the differentially expressed genes (DEGs) were identified for each cell cluster and the top 50 most significant DEGs were used to annotate each cluster with cell type name. We also sub-clustered cells within each lineage to identify transcriptomically distinct subpopulations.

### Cellular composition analysis of cell type:

We performed statistical tests for differences in cell type composition between pre-treatment samples and post-treated samples by using a Bayesian model-based tool https://github.com/theislab/scCODA ^46^.

### Differential expression analysis:

We identified DEGs for cell subpopulations of interest using Seurat which was filtered to select significant DEGs (log2 fold change > 1.0 or <−1.0 and FDR q-value < 0.05) between pre-treatment samples and post-treated samples. For pathway analysis, the curated gene sets (including Hallmark, GO, KEGG, REACTOME gene sets) were downloaded from the Molecular Signature Database, and Gene set enrichment analysis using fGSEA software package ^47^ was performed to identify significantly enriched signaling pathways (FDR q-value < 0.01) between pre-treatment samples and post-treated samples.

#### SLC38A1 Immunhistochemistry

IHC was performed on 5 μm thick paraffin sections following the standardized protocol^48^. Briefly, slides were deparaffinized in 3 changes of xylene for 5 minutes each, followed by rehydration through a graded series of alcohol. Antigen retrieval was performed in citrate buffer (pH 6.0) for 10 minutes, followed by subsequent cooling for 20 minutes and blocking of endogenous peroxidase with 30% hydrogen peroxide. Slides were next incubated with the primary antibody Anti-SLC38A1 (Millipore Sigma, catalog HPA052272, 1:25) at 4°C, overnight, washed 3 times with Tris-buffered saline (5 minutes each), and incubated with biotinylated anti-rabbit secondary antibody (1:200) for 1 hour at room temperature. After treating the slides with HRP conjugated ABC complex (Vectastain, Vector Laboratories) for 1 hour at room temperature, color was developed with DAB (Vector Laboratories), and slides were counterstained with hematoxylin, mounted with DPX, and examined under an Olympus DP73 microscope for imaging, analysis, and interpretation. Sections from a reactive LN served as a positive control, while those without the addition of a primary antibody served as negative controls.

### Statistical analysis:

Unless otherwise indicated, all calculations and statistical analyses were carried out using GraphPad Prism software v. 8 or 9. Figure legends indicate specific statistical analyses used. Statistically significant differences between 2 groups were assessed by unpaired Student t-tests. Ordinary one-way analysis of variance (ANOVA) was used to analyze more than 2 groups. Two-way ANOVA was used to analyze treatment responses between multiple groups. Results are expressed as mean ± standard deviation (SD) (as noted in the figure legends) of at least 3 independent experiments. Survival analysis for *in vivo* studies was done using Log-rank (Mantel–Cox) test. P-value was considered statistically significant with: *p: 0.05; **p: 0.01-0.0053; ***p: 0.001 - 0.0002; ****p < 0.0001.

## Figures and Tables

**Table 1:** Treatment outcome

	N (%)
**Objective response**	
**CR**	5 (18)
**mCR +/− HI**	10 (36)
**mCR with HI**	4
**mCR without HI**	6
**HI alone**	3 (11)
**No response**	10 (36)
**Proceeded to allogeneic SCT**	7
**Early mortality**	
**30-day**	0
**60-day**	1 (3.6%)

Abbreviations: N-number; CR-complete response; mCR-manow clinical response; HI-hematological improvement; SCT-stem cell transplantation.
